# Urinary exosomes reveal protein signatures in hypertensive patients with albuminuria

**DOI:** 10.18632/oncotarget.17787

**Published:** 2017-05-11

**Authors:** Laura Gonzalez-Calero, Paula J. Martínez, Marta Martin-Lorenzo, Montserrat Baldan-Martin, Gema Ruiz-Hurtado, Fernando de la Cuesta, Eva Calvo, Julian Segura, Juan Antonio Lopez, Jesús Vázquez, Maria G. Barderas, Luis M. Ruilope, Fernando Vivanco, Gloria Alvarez-Llamas

**Affiliations:** ^1^ Department of Immunology, IIS-Fundacion Jimenez Diaz, REDinREN, Madrid, Spain; ^2^ Department of Vascular Physiopathology, Hospital Nacional de Paraplejicos SESCAM, Toledo, Spain; ^3^ Hypertension Unit, Instituto de Investigación Imas12, Hospital Universitario 12 de Octubre, Madrid, Spain; ^4^ Ibermutuamur, Madrid, Spain; ^5^ Laboratory of Cardiovascular Proteomics CNIC, Madrid, Spain; ^6^ Department of Biochemistry and Molecular Biology I, Universidad Complutense, Madrid, Spain

**Keywords:** exosomes, hypertension, albuminuria, renin-angiotensin system, proteomics

## Abstract

Albuminuria is an indicator of cardiovascular risk and renal damage in hypertensive individuals. Chronic renin–angiotensin system (RAS) suppression facilitates blood pressure control and prevents development of new-onset-albuminuria. A significant number of patients, however, develop albuminuria despite chronic RAS blockade, and the physiopathological mechanisms are underexplored. Urinary exosomes reflect pathological changes taking place in the kidney. The objective of this work was to examine exosomal protein alterations in hypertensive patients with albuminuria in the presence of chronic RAS suppression, to find novel clues underlying its development. Patients were followed-up for three years and were classified as: a) patients with persistent normoalbuminuria; b) patients developing *de novo* albuminuria; and c) patients with maintained albuminuria. Exosomal protein alterations between groups were identified by isobaric tag quantitation (iTRAQ). Confirmation was approached by target analysis (SRM). In total, 487 proteins were identified with high confidence. Specifically, 48 proteins showed an altered pattern in response to hypertension and/or albuminuria. Out of them, 21 proteins interact together in three main functional clusters: glycosaminoglycan degradation, coagulation and complement system, and oxidative stress. The identified proteins constitute potential targets for drug development and may help to define therapeutic strategies to evade albuminuria progression in hypertensive patients chronically treated.

## INTRODUCTION

Hypertension is a major risk factor for cardiovascular and renal disease. In patients with primary hypertension and normal renal function, increased urinary albumin excretion (albuminuria) is a marker of cardiovascular disease progression and of renal function worsening [1–3]. Its predictive capacity is based on the fact that microvascular damage is present in the kidney and this and other forms of target organ damage have been shown to predict mortality even when global cardiovascular risk (SCORE-based evaluated) is not elevated [4]. Chronic suppression of the renin–angiotensin system (RAS) has been shown to facilitate blood pressure (BP) control and prevent the development of new-onset albuminuria [5]. Nonetheless, we and others have shown that hypertensive patients under chronic RAS suppression may present albuminuria; in particular, *de novo* albuminuria developed in up to 16.1% of normoalbuminuric patients during a three–year period of follow-up [6, 7]. The physiopathological mechanisms mediating albuminuria progression in hypertensive patients under chronic RAS suppression are, however, underexplored.

Exosomes are biological messengers with proven roles in regulating immune response, antigen presentation, RNA and protein transfer, and cell–cell interaction/signaling. Exosomes from the renal system may provide with novel clues in the clinical setting of albuminuria [8, 9, 10]. Indeed, urinary exosomes contain proteins characteristic of every renal tubule epithelial cell type and from the urinary collecting system, including proteins that are characteristic of the membrane and cytoplasm of the cells in which they have been generated. As such, they should be regarded not only as waste disposal units, but also as messengers, carrying molecular indicators of renal dysfunction and structural injury [11]. This role for exosomes gains particular importance in complex scenarios where multi-organ crosstalk takes place [8, 12]. We previously showed that urinary exosomes reflect protein changes taking place in the kidney in diabetic nephropathy [13], thus constituting an accessible source of “information” compared to renal biopsy. Additionally, exosomes constitute a sub-proteome of the urine and its analysis allow approaching molecular changes that otherwise are hidden if pursued in the whole urine. Previous studies from our group showed molecular fingerprints in urine and plasma linked to albuminuria development in hypertensive patients who are under chronic RAS suppression. In this study, we investigate a potential role for urinary exosomes in the search for novel markers in this clinical setting.

## RESULTS

### Exosomal proteins identified in hypertensive patients under chronic RAS suppression

Urinary exosomes and their potential role in the physiopathological mechanisms responsible for albuminuria development in hypertensive patients under chronic RAS suppression were investigated. Quantitative iTRAQ-LC-MS/MS proteomic analysis was used to explore exosomal protein alterations, allowing deep coverage of the analyzed proteome as previously shown in plasma in this clinical setting [14]. The present work focused on proteins. While intact endogenous peptides are also of great interest, and for which different analytical strategies should be applied, they were beyond the scope of the present study. Urine exosome samples from the 23 subjects (5 control and 18 hypertensive patients) were used for iTRAQ analysis. Two biological replicates were analyzed per group with each comprising 2–4 individual samples (see Supplementary Figure 1 for experimental design). Proteomic data derived from this study are deposited in Peptide-Atlas (accession number PASS00970).

A total of 487 proteins with more than 3 peptides were identified in urinary exosomes (Supplementary Table 1). Among these, 100 proteins were identified for the first time in urinary exosomes according to the ExoCarta database (Supplementary Table 2). Exosomal markers including Tsg101, CD63, annexins A3, A4, A5, A6, and A7, flotillin-1, clathrin1, and LAMP1 were identified and no significant differences were found between N, dnA and MHA groups confirming un-biased exosomal isolation. Some of the identified proteins have been previously assigned specifically to Bowman's capsule, proximal tubule, loop of Henle, distal tubule, collecting duct or bladder, and no differences in response to albuminuria or hypertension were detected (Supplementary Table 3).

### Exosomes have distinct protein signatures in response to albuminuria

Proteins whose levels were identified to be altered were those with a significance cut off value of Zq ≥ 1.4 (absolute value) versus the control group, and showing intra-group consistency of variation between the two biological replicates analyzed per group. With these criteria, iTRAQ quantitative analysis revealed 48 proteins (out of the 487 identified) showing altered levels in hypertensive patients under chronic RAS suppression as compared with healthy subjects (Table 1). The magnitude of protein alteration in Table 1 is represented by a heat map (red, up-regulation; green, down-regulation *versus* the control group). Three main trends could be observed, being up- or down-regulated in response to: hypertension without albuminuria (9 proteins), maintained albuminuria (18 proteins), and *de novo* albuminuria (21 proteins). According to the Human Protein Atlas, 56% of the identified proteins have annotated expression in the kidney, mainly in the tubule (26 proteins) but also in the glomerulus (13 proteins). The majority of these proteins were also identified in a recent study characterizing the proteome of urinary exosomes specifically released by podocytes [15].

**Table 1:** Proteins identified in urinary exosomes from hypertensive patients under chronic RAS suppression showing significantly altered levels in response to albuminuria

Fasta	Gene	Description	ID Pept1	Evidence in kidney (HPA,refs)	Zq N	Zq dnA	Zq MHA
**Proteins responding to maintained albuminuria**							
P01859	IGHG2	Ig gamma-2 chain C region	11	-			
P02750	LRG1	Leucine-rich alpha-2-glycoprotein	11	T			
P04217	A1BG	Alpha-1B-glycoprotein	11	-			
P25311	AZGP1	Zinc-alpha-2-glycoprotein	10	T			
P15586	GNS	N-acetylglucosamine-6-sulfatase	6	G,T			
P04066	FUCA1	Tissue alpha-L-fucosidase	5	T			
P34059	GALNS	N-acetylgalactosamine-6-sulfatase	5	G,T			
Q9BTY2	FUCA2	Plasma alpha-L-fucosidase	4	T			
P07355	ANXA2	Annexin A2	36	G,T			
P04083	ANXA1	Annexin A1	29	G			
P10253	GAA	Lysosomal alpha-glucosidase	30	T			
P01011	SERPINA3	Alpha-1-antichymotrypsin	14	-			
P08236	GUSB	Beta-glucuronidase	14	G,T			
P15289	ARSA	Arylsulfatase A	11	G,T			
P00747	PLG	Plasminogen	8	T, I [57]			
P51688	SGSH	N-sulphoglucosamine sulphohydrolase	5	T			
B9A064	IGLL5	Immunoglobulin lambda-like polypeptide 5	5	T			
P05543	SERPINA7	Thyroxine-binding globulin	11	-			
**Proteins responding to de novo albuminuria (earlier response)**							
P00450	CP	Ceruloplasmin	35	PECs [58]			
P01024	C3	Complement C3	57	PT, I [59]			
P01009	SERPINA1	Alpha-1-antitrypsin	32	T			
P02787	TF	Serotransferrin	31	PTECs [60]			
P49221	TGM4	Protein-glutamine gamma-glutamyltransferase 4	16	-			
P0C0L4	C4A	Complement C4-A	41	T [59]			
P16278	GLB1	Beta-galactosidase	14	G,T			
P01857	IGHG1	Ig gamma-1 chain C region	14	-			
A2BHY4	C4B-1	Complement component C4B	5	T [59]			
P01620		Ig kappa chain V-III region SIE	4	-			
Q96P63	SERPINB12	Serpin B12	7	T			
P07339	CTSD	Cathepsin D	13	G,T			
Q6UX06	OLFM4	Olfactomedin-4	21	T [61]			
P01008	SERPINC1	Antithrombin-III	8	G,T			
P02511	CRYAB	Alpha-crystallin B chain	7	G,T			
P05164	MPO	Myeloperoxidase	11	inflitrates [62]			
P08571	CD14	Monocyte differentiation antigen CD14	16	-			
P00915	CA1	Carbonic anhydrase 1	9	-			
O00187	MASP2	Mannan-binding lectin serine protease 2	9	G,T			
P02730	SLC4A1	Band 3 anion transport protein	8	T			
P05090	APOD	Apolipoprotein D	7	-			
Proteins responding to hypertension with normoalbuminuria								
P05154	SERPINA5	Plasma serine protease inhibitor	21	T			
P05109	S100A8	Protein S100-A8	18	inflitrates [63]			
P06702	S100A9	Protein S100-A9	15	inflitrates [63]			
Q6UVK1	CSPG4	Chondroitin sulfate proteoglycan 4	4	G,T			
P12277	CKB	Creatine kinase B-type	12	-			
P01133	EGF	Pro-epidermal growth factor	26	T			
P01023	A2M	Alpha-2-macroglobulin	18	G,T			
Q6UX73	C16orf89	UPF0764 protein C16orf89	4	T			
Q14624	ITIH4	Inter-alpha-trypsin inhibitor heavy chain H4	14	T			

Colour-grade scale refers to the magnitude of the alteration *versus* the control group: red, up-regulation; green, down-regulation.

Differentially expressed proteins were those identified with more than three peptides and Zq ≥ 1.4 (absolute value) *versus* control group (*p*-value ≤ 0.05). Zq denotes the mean for the two biological replicates analyzed per group. ^1^Identified peptides by LC-MS/MS. G: glomerulus; T: tubule; -: no data; PTECs (proximal tubular epithelial cells); I (interstitium); PECs (parietal epitheilal cells); PT (proximal tubule). Annotated expression in kidney: Human Protein Atlas (HPA).

**Figure 1:**
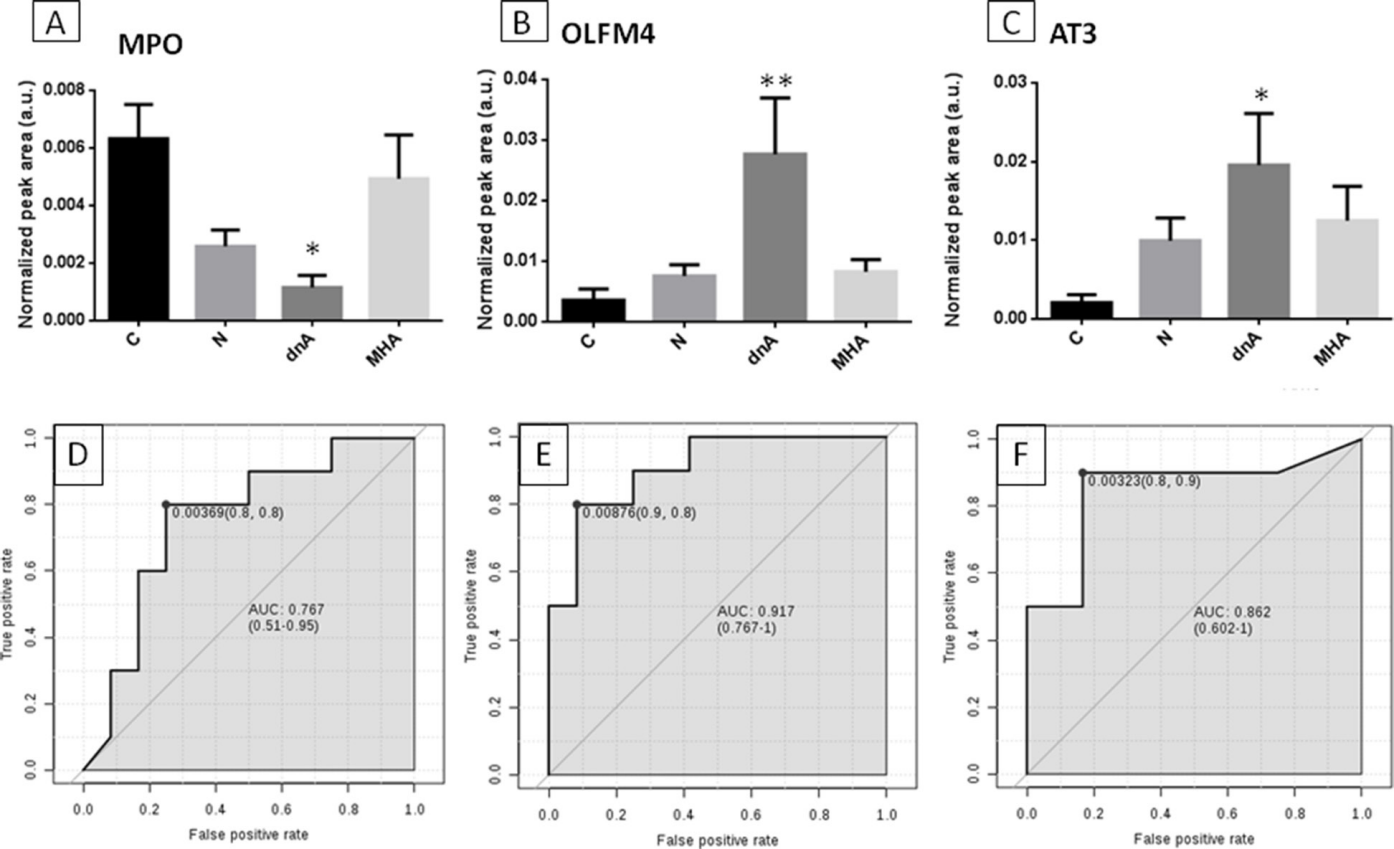
Confirmation of proteins variation by target analysis SRM-LC-MS/MS analysis confirmed previous variations identified by iTRAQ for three representative proteins showing significant alteration in response to dnA (early response): MPO (**A**), OLFM4 (**B**) and AT3 (**C**). Non-parametric Kruskal-Wallis test with Dunn's multiple comparisons pos*t*-test and 95% confidence level was performed between each pathological condition (N, dnA, MHA) and control (C). (Panels **D** to **F)** show receiver operating curves (ROC) for these proteins, respectively; *t-test* values were 0.029 (MPO), 0.0089 (AT3) and 0.0097 (OLFM4).

We evaluated a sub-set of representative proteins significantly responding to *de novo* albuminuria, myeloperoxidase (MPO), olfactomedin-4 (OLFM4) and antithrombin-III (AT3), using a different analytical technique (SRM-LC-MS/MS) in an independent cohort of 40 non-diabetic hypertensive patients under chronic suppression of RAS and in 14 healthy subjects. In this target analysis, all subjects who were recruited for the iTRAQ analysis (but two control individuals) were included, and individual samples (not pools) were analyzed. A specific SRM assay was developed with high sensitivity and high-throughput capacity in alignment with our previously published study in urine [16]. Changes in their levels were confirmed for three representative proteins, OLFM4, AT3 and MPO, chosen in view that they showed variation in dnA, which is an earlier response in the pathological process, their variation rate, their abundance (estimated in view of number of identified peptides) or their relationship with other proteins previously identified in plasma/urine in this clinical context (e.g. ceruloplasmin or MMP9). OLFM4 and AT3 levels were increased and MPO was decreased in the dnA group. Receiver operating curves showed good sensitivity and specificity in differentiating the dnA group from the control group, with *t-test* values of 0.029 (MPO), 0.0089 (AT3) and 0.0097 (OLFM4) and area under the curve (AUC) values were 0.767, 0.862 and 0.917, respectively (Figure 1).

### Biological response and protein interaction networks

**Figure 2:**
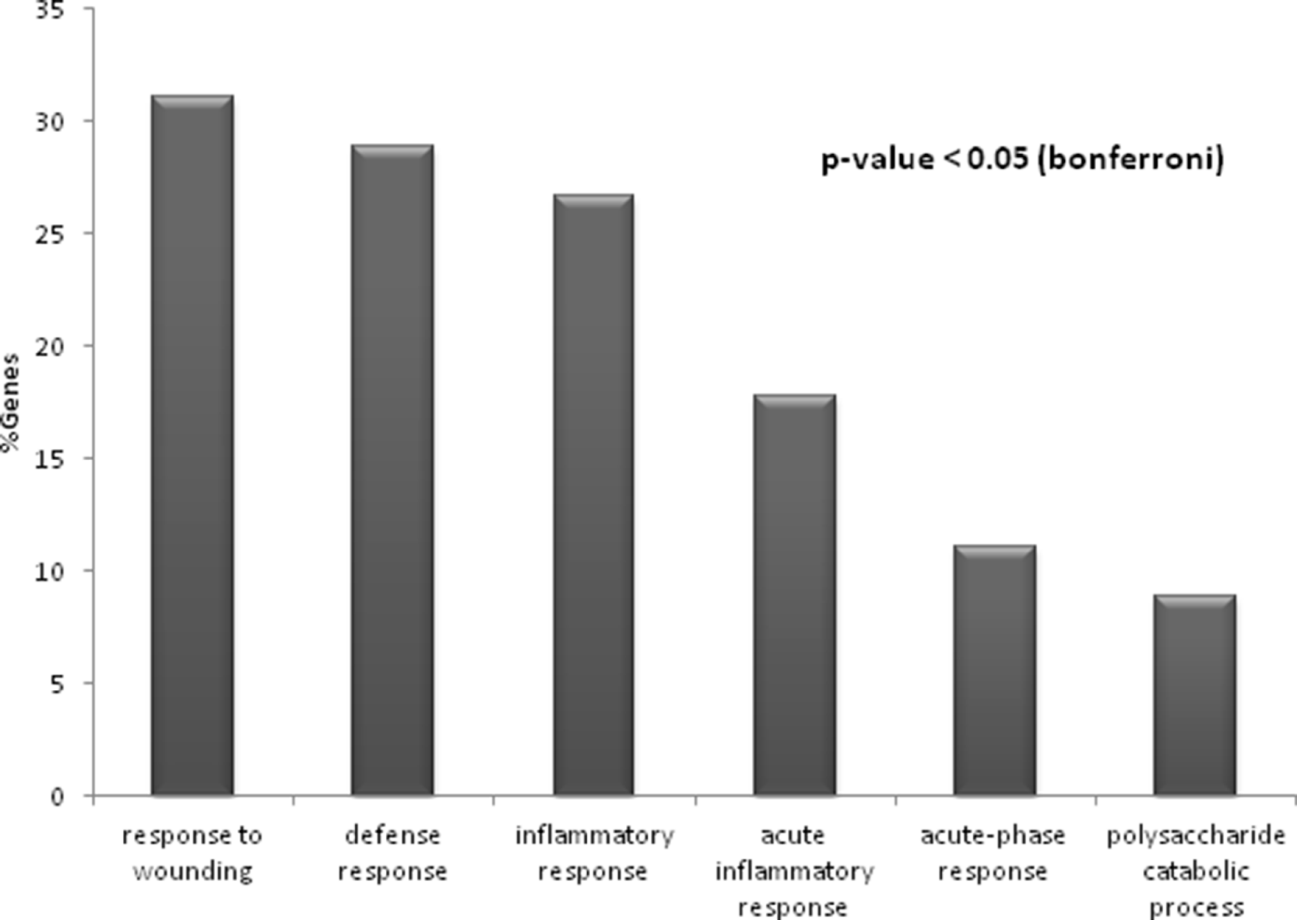
Significantly enriched processes (*p*-value < 0.05) identified for the entire set of 48 altered proteins in response to hypertension and albuminuria The percentage of genes associated with each biological process is represented.

**Figure 3:**
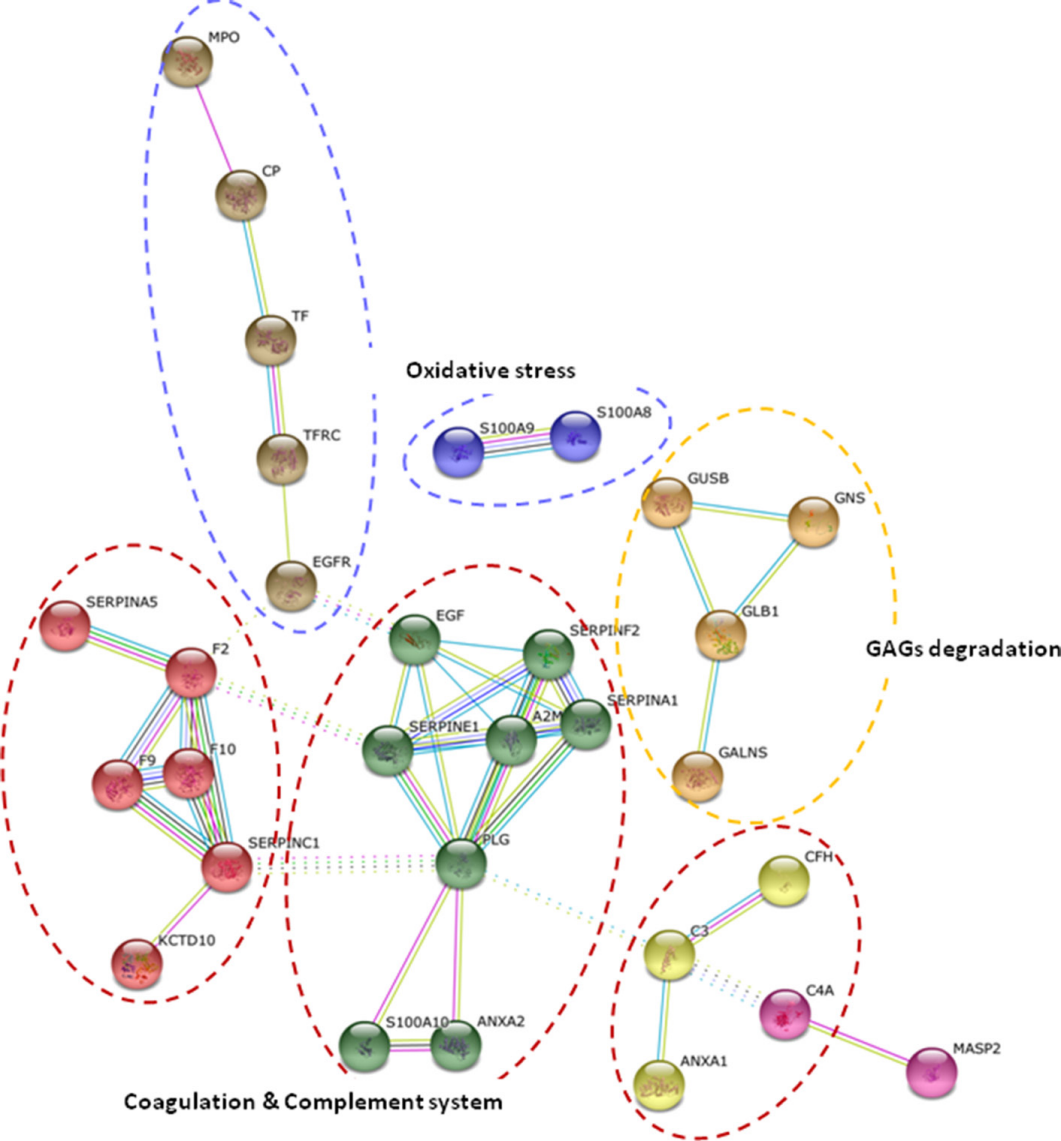
Functional analysis of protein variations String pathway analysis revealed three main clusters of interaction, pointing to glycosaminoglycan (GAG) degradation, coagulation and complement system, and oxidative stress as main physiopathological processes underlying hypertension under chronic RAS suppression and albuminuria development.

Significantly enriched GO terms (*p-value* < 0.05) of the main biological processes identified from protein alterations in response to hypertension and albuminuria progression revealed the involvement of inflammatory response, defense and wounding (Figure 2). In particular, the altered proteins are major players in immune response, complement activation, proteolysis and metabolic processes, mainly related to metabolism of sulphur compounds, lipids and polysaccharides. Protein-protein interaction networks revealed that 21 out of those 48 proteins interacted together, and are implicated in three main functional clusters: “glycosaminoglycan (GAG) degradation”, “coagulation and complement system”, and “oxidative stress” (graphically summarized in Figure 3).

## DISCUSSION

Hypertension is associated with end-organ damage, particularly affecting heart, vessels and kidney, leading to significant worsening of cardiovascular and renal prognosis. Albuminuria is an indicator of cardiovascular risk and of end-stage renal disease. We recently reported significant alterations in proteins from urine and plasma in response to albuminuria and its *de novo* development in these hypertensive patients [14, 16, 17], demonstrating that subjacent biological mechanisms have a measurable molecular phenotype in biological fluids which, in some cases, may allow for anticipating the response of the traditional clinical marker (albuminuria). Exosomes have been shown to reflect molecular changes taking place in kidney tissue from patients with diabetic nephropathy [13]. In the present study we searched for the strongest variations in urinary exosomes linked to albuminuria, in search of a better understanding of the interconnected dynamic processes taking place in the non-diabetic hypertensive kidney under chronic RAS suppression.

### Glomerular basement membrane disruption, proteases inhibition and inflammatory response

**Figure 4:**
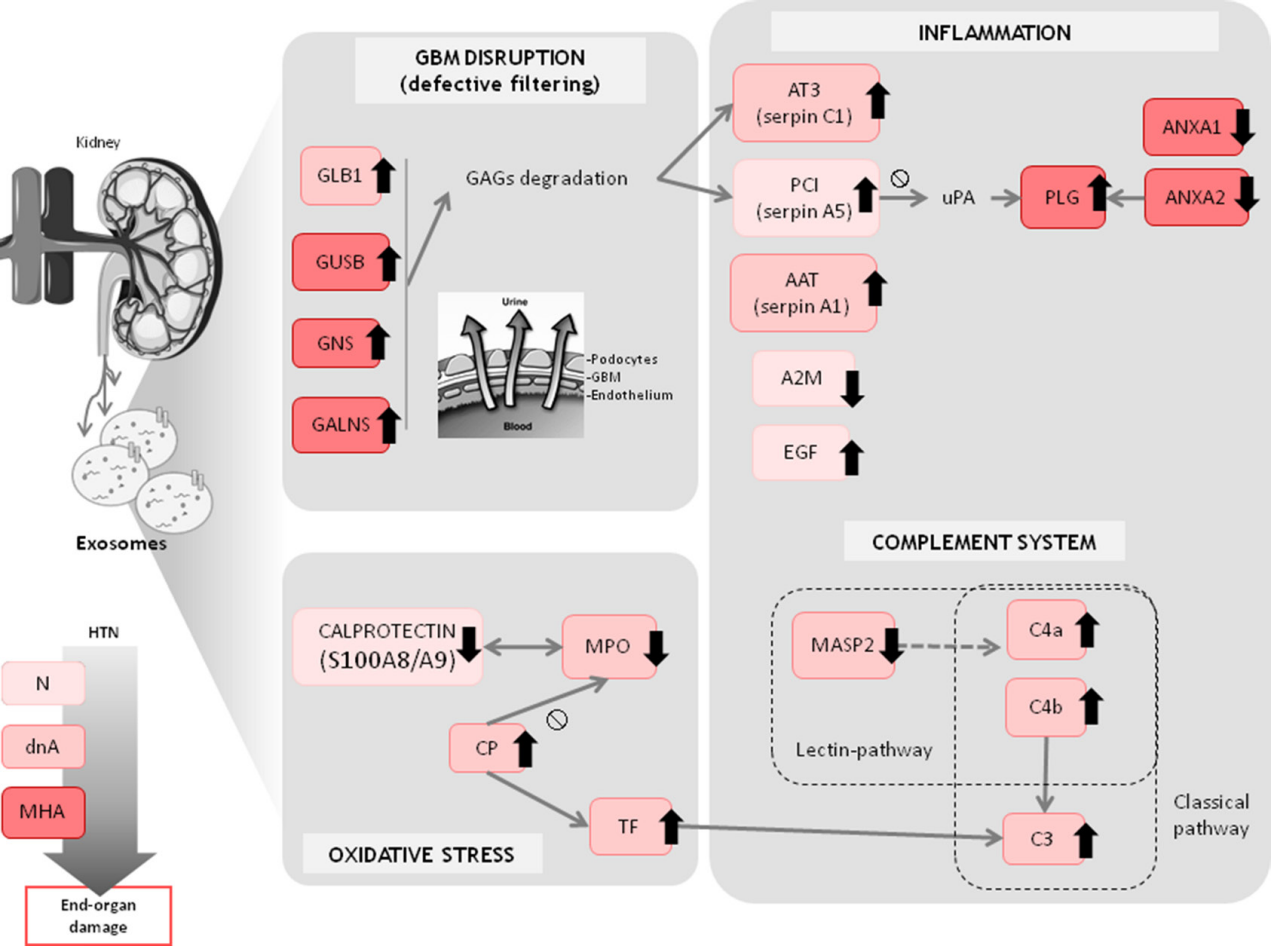
Overview of main altered protein patterns identified in exosomes from hypertensive patients under chronic RAS suppression developing albuminuria Trends refer to control group. Analysis of urinary microvesicles allows a closer view of the changes potentially taking place in the kidney that otherwise could not be accessed non-invasively.

Enzymes linked to the GAG pathway, specifically beta-galactosidase (GLB1), beta-glucuronidase (GUSB), N-acetyl-glucosamine-6-sulfatase (GNS) and N-acetyl-galactosamine-6-sulfatase (GALNS), were increased in the MHA group, and GLB1 was also increased in the dnA group (Figure 4). These four enzymes are responsible for the GAG degradation and may be related to glomerular basement membrane (GBM) disruption, which could lead to defective filtering [18]. One of the degradation products that would be enhanced in pathologic conditions is the proteoglycan heparan sulphate. Previous findings suggest that heparan sulphate facilitates the passage of proteins through the GBM [19]. This proteoglycan binds to and modulates the effects of several proteins, including heparin-dependent serine protease inhibitors such as antithrombin III (AT3, SerpinC1) and protein C inhibitor (PCI, PAI-3, SerpinA5) [20], both of which we found increased in urinary exosomes of hypertensive patients. AT3 is related to homeostasis in the kidney, and its concentration in urine correlates with albumin and plasminogen (PLG) excretion [21]. The main source of PCI is liver; however, its synthesis has also been reported in kidney where it is produced mainly by proximal tubular epithelial cells (PTECs) [22]. PCI is a pro-coagulant and pro-inflammatory protein that inhibits urinary plasminogen activator (uPA), a serine protease that activates plasminogen by converting it to plasmin, which acts to lyse fibrin clots. PLG is synthesized in liver but often extravasates during inflammation. We also found increased PLG in urinary exosomes from the MHA group. PLG can bind to annexin A2 (ANXA2), promoting its activation to form plasmin [23]. We found decreased levels of exosomal ANXA2 and ANXA1 in the MHA group. Annexins are a family of calcium-dependent phospholipid-binding proteins with anti-inflammatory activity [24]. Down-regulation of both ANXA1 and ANXA2 exosomal proteins is in agreement with a pro-inflammatory scenario in this clinical context.

Two exosomal proteins whose levels were altered in the normoalbuminuric (N) group as compared with controls are also involved in coagulation and inflammatory response: epithelial growth factor (EGF), which was increased, and a-2 macroblobulin (A2M), which was decreased. EGF in the kidney is a modulator of glomerular dynamics, renal metabolism and tubular transport, and plays a role in maintaining and repairing the epithelium [25]. A2M is a major inhibitor of proteases in body fluids and is the most important inhibitor of circulating metalloproteinases (MMPs) [26]. The response of the two proteins, displaying alterations at an early stage when albuminuria is not present, point to subjacent de-regulation of renal metabolism. The decrease in the level of A2M in the N group, together with the up-regulation observed for the GAG degradation pathway in albuminuria are in agreement with a deleterious remodeling of the GBM involving pathological MMP-9 activity, which might contribute to glomerular hyperfiltration, albuminuria escape and loss of renal function, as previously published by us [27].

The level of alpha-1 antitrypsin (AAT, SerpinA1), a heparin-independent serine protease inhibitor, was significantly increased in exosomes from albuminuric patients, which is consistent with our previous finding that AAT levels are increased in whole urine from hypertensive patients who develop albuminuria [16]. AAT has also been suggested to regulate inflammation and the accumulation of mesangial matrix, protecting the urinary tract against attack by proteases, and could be a marker of podocyte stress [28].

### Complement system and inflammatory response

A sub-set of the identified proteins is linked to the complement system, as part of the immune response [29]. The complement system has three activation pathways: the classical, alternative, and lectin pathways. The level of mannose-binding lectin (MBL)-associated serine protease-2 (MASP-2) was decreased in exosomes from the dnA group, while components C3, C4a and C4b were increased in albuminuria condition, pointing to a disfavored lectin pathway. Levels of complement C3 and C4 are also increased in chronic inflammation [30]. Moreover, excess protein traffic occurs in proteinuric nephropathies, causing an inflammatory response with local increases in C3 [31], and high baseline levels of C3 correlate with higher risk of developing hypertension, diabetes mellitus or myocardial infarction [30]. Our data are in accord with these previous observations.

### Oxidative stress

We detected an increase in the levels of transferrin (TF) in exosomal fractions of hypertensive patients with albuminuria. Transferrin is an iron-binding serum protein that upregultaes C3 synthesis in PTECs [32]. The iron moiety of transferrin is toxic to PTECs as a result of intracellular iron accumulation and peroxidative injury after reabsorption. Conversely, iron depletion was shown to have a positive effect in inhibiting oxidative stress in experimental diabetes [33]. Our data indicate an accumulation of transferrin in kidney in albuminuria, with a potential deleterious effect *via* excess iron accumulation. Ceruloplasmin (CP) levels were also found increased in patients with hypertension and albuminuria. CP is a copper-containing glycoprotein antioxidant that may function as a pro-oxidant in conditions of increased oxidative stress by inducing ROS production and low-density lipoprotein oxidation [34]. We detected a positive correlation between CP levels in urinary exosomes and increased albuminuria, as we previously described for plasma CP from hypertensive patients under chronic RAS suppression who progress to albuminuria [17].

CP also acts by binding to and inhibiting the activity of myeloperoxidase (MPO) [35], the exosomal levels of which were decreased in the dnA group. Following the same trend than observed for MPO in the N and dnA groups were two intracellular calcium-binding proteins, S100A8 and S100A9, which form the heterodimer calprotectin. MPO and calprotectin have been shown to work synergistically to stimulate ROS formation [36]. Additionally, calprotectin also inhibits MMPs, whose activities were previously found to be increased in this clinical setting [27]. While an increase in the level of blood S100 proteins in response to inflammation has been widely documented and positively correlate with cardiovascular risk, diabetes and proteinuria [37, 38], the opposite was found here for exosomal fractions in hypertensive patients with incipient or no albuminuria, which showed decreased levels of S100A8 and S100A9. This may be explained on the basis of tissue repair mechanisms. In kidney disease, controversy exists over the beneficial/deleterious effect of M1/M2 macrophage polarization. In one study, a shift in macrophage polarization from M1 to M2 was related to a decrease in albuminuria [39], while in another study, repolarization to M1 by drug treatment inhibited kidney damage progression [40]. In an ischemia-reperfusion-induced injury model, mice genetically deficient for calprotectin presented an increase in M2-polarized macrophages during the repair phase, leading to ineffective tissue repair, increased renal damage and sustained inflammation [41]. This tissue repair effect may require physical contact between the M2 macrophage and the injured tubular epithelial cell [42], which we hypothesize could be mediated by exosomes. Our data are in agreement with an inefficient tissue repair at early stages in albuminuria development and maintenance.

This is an initial study that focuses on a largely unexplored clinical field. We have identified protein changes in urinary exosomes from hypertensives developing albuminuria, despite of chronic RAS suppression with different variation trends. In particular, some proteins show a dynamic change in the sense that they respond to dnA and apparently return to normalized levels in MHA. This finding, also observed in specific metabolites as shown in previous studies, is not surprising and we can hypothesized that the mechanisms operating when the conditions for a progression of albuminuria are present are reset once albuminuria is more established. The main limitation of the present study is the low number of patients and thus the clinical usefulness of the exosomal proteins identified and the proposed physiological changes taking place linked to albuminuria development in hypertensive patients under chronic suppression of RAS should be validated in larger multicenter cohorts. Although unavoidable, a potential bias regarding patients’ medication compared to control individuals could be a limitation of the study. However, particular attention was paid to protein changes with albuminuria development and no significant differences were observed between patients’ groups. All them were under RAS suppression and other drugs, if necessary. As a strength, we would highlight that this is the first study to explore exosomes as a novel source of diagnostic/prognostic markers for albuminuria development in this context. The present findings complement and extend our previous work in urine, plasma and circulating extracellular vesicles. We believe that exosomes contain clues to understand the diverse progression of hypertension under chronic treatment, and our data indicates that this issue should be investigated more in depth.

## MATERIALS AND METHODS

### Patient selection

**Figure 5:**
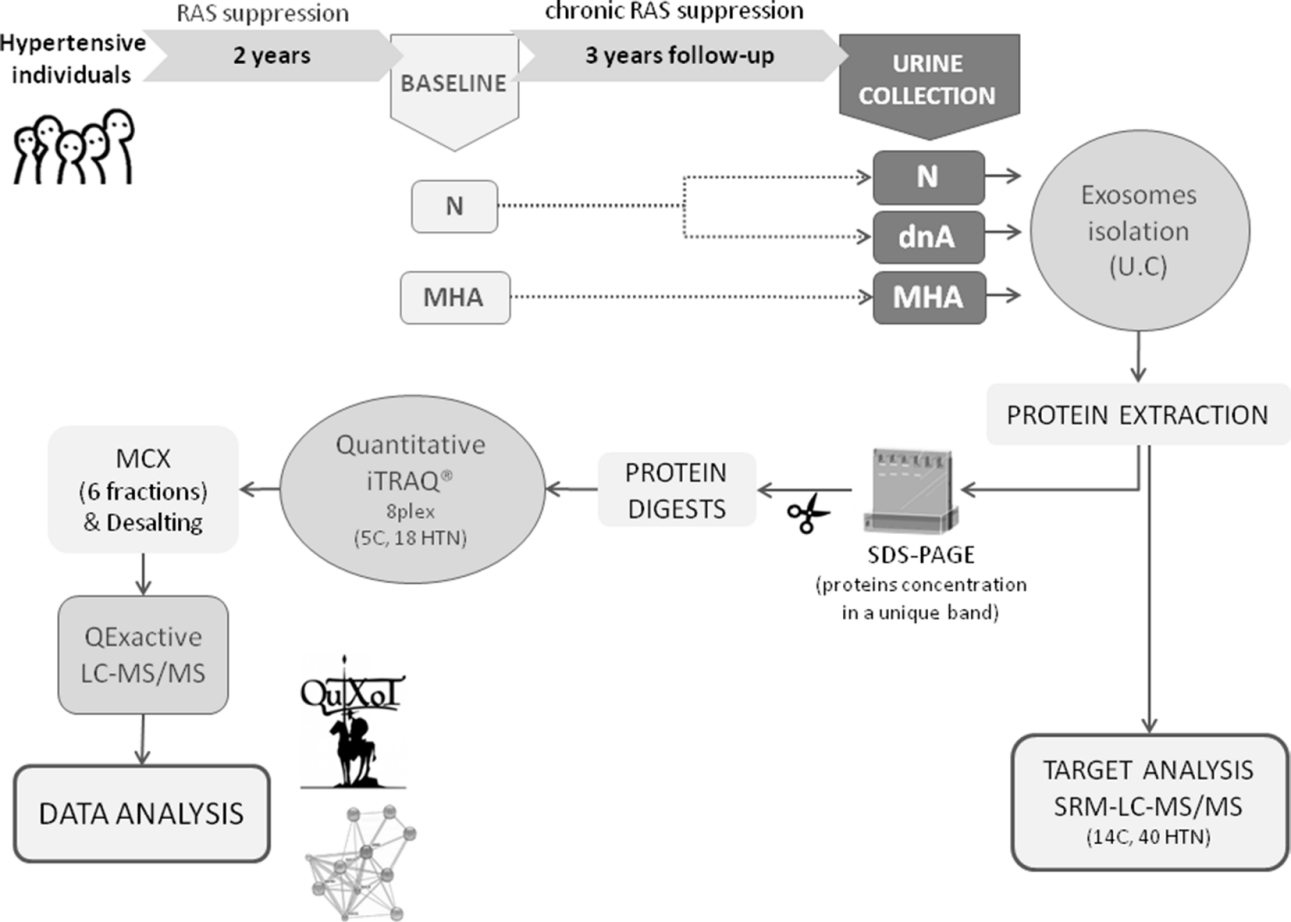
Schematic representation of the study workflow Urine samples from 40 non-diabetic hypertensive patients and 16 healthy controls were collected. In iTRAQ experiment 5 control samples and 18 patients, 6 of each group, were included (6 N, 6 dnA and 6 MHA). By SRM, samples from the 40 hypertensives and from 14 healthy controls were analyzed individually. C: control, N: normoalbuminuria, dnA: *de novo* albuminuria, MHA: maintained albuminuria, UC: ultracentrifugation, SRM: selected reaction monitoring, MCX: mixed-cation exchange, HTN: hypertension.

A schematic workflow is shown in Figure 5. Patient selection was based on a previous study showing the development of *de novo* albuminuria in hypertensive patients during chronic RAS suppression [7]. In that study, the evolution of patients from the Hypertension Unit-Hospital 12 de Octubre who had been under chronic RAS suppression for at least five years was reviewed. Baseline data were obtained after a 3-month stabilization period. From baseline, patients were subsequently followed for a minimum period of three years, during which time progression to albuminuria was evaluated. RAS suppression was maintained during the entire length of follow-up. Of the normoalbuminuric patients at baseline, 16.1% developed *de novo* albuminuria (i.e., absent at baseline and developed later) during the 3-year period. *De novo* albuminuria development was defined as either new-onset high albuminuria (albumin-to-creatinine ratio 20–200 mg/g of creatinine in men and 30–300 mg/g of creatinine in women) confirmed in at least a second occasion among the 6-monthly determinations performed in three samples of early-morning urine, or very high albuminuria (> 200 mg/g creatinine in men and > 300 mg/g creatinine in women). We performed exosomal analysis at the end of the third year of follow-up. A group of 40 non-diabetic hypertensive patients were selected as a representative cohort and classified into the following three sub-groups: a) patients who remained normoalbuminuric during the follow-up (defined as N); b) patients developing *de novo* albuminuria during the follow-up (defined as dnA); and c) patients with persistent albuminuria since baseline and during the follow-up (defined as MHA, maintained high albuminuria). Table 2 lists the baseline characteristics and medication of patients. The presence of secondary forms of arterial hypertension and diabetes mellitus were excluded. A control group of 16 healthy normotensive subjects (defined as C) was also included (Table 3). The study fulfilled the requirements of an “Omics” study in terms of group size and technical workflow [43]. The clinical study was approved by the Ethics Committee of the Hospital 12 de Octubre and was conducted according to the principles of the Declaration of Helsinki. All patients signed written informed consent before inclusion.

**Table 2:** Baseline characteristics and medication of patients

Baseline characteristics	*N* (*n* = 19)	dnA (*n*= 11)	MHA (*n*= 10)	*p*-value
**Age (years)**	61 ± 9	65 ± 9	63 ± 9	0.362
**Total cholesterol (mg/dl)**	197 ± 34	170 ± 13	187 ± 21	0.032
**Triglycerides (mg/dl)**	121 ± 50	106 ± 50	120 ± 39	0.34
**HDL cholesterol (mg/dl)**	53 ± 12	50 ± 17	49 ± 9	0.493
**LDL cholesterol (mg/dl)**	120 ± 33	97 ± 14	115 ± 21	0.056
**Glycemia (mg/dl)**	105 ± 12	99 ± 9	100 ± 12	0.321
**Uric acid (mg/dl)**	5.1 ± 1.8	6.1 ± 1.6	5.7 ± 1.5	0.373
**eGFR (ml/min/1.73m**^2^)	88 ± 11	81 ± 17	79 ± 20	0.464
**Systolic blood pressure (mmHg)**	137 ± 15	131 ± 20	137 ± 19	0.616
**Diastolic blood pressure (mmHg)**	83 ± 9	77 ± 10	84 ± 12	0.273
**ACR (mg/g)**	8 ± 7	83 ± 38	125 ± 125	< 0.0001
**Medications**				
**Antihypertensives, %**				
** ACEi**	11	9	30	0.316
** ARB**	67	64	70	0.849
** Diuretic**	43	36	50	0.793
** Calcium channel blocker**	33	18	60	0.148
** Beta blocker**	19	18	60	0.061
**Other treatments, %**				
** Anticoagulant agent**	24	27	20	0.916
** Lipid lowering agents**	68	55	80	0.467
** Antidiabetic agent**	0	0	0	> 0.9999
** Antialdosteronics**	21	18	10	0.761
** Hypouricemics**	0	18	0	0.067

Values expressed as mean ± SD or percentages (%). BMI: body mass index; HDL: high-density lipoprotein cholesterol; LDL: low-density lipoprotein cholesterol; N: normoalbuminuria; dnA: de novo albuminuria; MHA: maintained high albuminuria; ACR: albumin creatinine ratio; eGFR: estimated glomerular filtration rate.

**Table 3:** Baseline characteristics of the control group

Baseline characteristics of healthy subjects	
**Age (years)**	56 ± 7
**Total cholesterol (mg/dl)**	199 ± 38
**Triglycerides (mg/dl)**	83 ± 33
**HDL cholesterol (mg/dl)**	62 ± 19
**LDL cholesterol (mg/dl)**	119 ± 31
**Glycemia (mg/dl)**	91 ± 13
**Uric acid (mg/dl)**	4.4 ± 1.3
**eGFR (ml/min/1.73m2)**	93 ± 14
**ACR (mg/g)**	4 ± 3
**Systolic blood pressure (mmHg)**	< 140
**Diastolic blood pressure (mmHg)**	< 90

HDL: high-density lipoprotein cholesterol; LDL: low-density lipoprotein cholesterol; ACR: albumin creatinine ratio; eGFR: estimated glomerular filtration rate.

### Urine collection and exosome isolation

Urine collection and exosome isolation based on ultracentrifugation were performed as described [13]. Briefly, morning urine was collected in a sterile container and stored at −80°C until analysis. For exosome isolation, urine was defrosted at 37°C and vigorously vortexed centrifuged at low-speed (17000 g, 10 min, 4°C), the supernatant was collected and the pellet was treated with 200 mg/mL dithiothreitol (DTT) in isolation solution (10 mM triethanolamine, 250mM sucrose, pH 7.6), heated at 37°C for 10 min to reduce Tamm-Horsfall polymeric protein, releasing entrapped exosomes and increasing exosomal recovery [13, 44, 45]. After a new low-speed centrifugation (17000 g, 10 min) the resulting pellet was discarded and the supernatant was pooled together with the supernatant from the first centrifugation step. This was then centrifuged at 175000 g for 70 min at 4°C to pellet the exosomes. Isolated exosomes were characterized by electronic microscopy. The typical shape of exosomes (double membrane, cup-shaped vesicles) and appropriate size in the range of 50–100 nm was observed [45].

### Identification of exosomal target proteins in hypertensive patients under chronic RAS suppression: iTRAQ-LC-MS/MS differential analysis

From the 40 non-diabetic hypertensive patients and 16 healthy controls referred above, urine samples from 5 control subjects and 18 hypertensive patients were used for exosome extraction and quantitative differential analysis by liquid chromatography coupled with tandem mass spectrometry (LC-MS/MS). Patients were classified into N (*n* = 6), dnA (*n* = 6) and MHA (*n* = 6) groups. Isobaric tags for relative and absolute quantification (iTRAQ, 8-plex) labelling was performed as described [14]. Two biological replicates (pools) were analyzed per group (C, N, dnA and MHA), with each comprising 2–4 individual samples (Supplementary Figure 1). One hundred micrograms of total protein from each biological replicate was loaded onto an SDS-PAGE gel to concentrate all the proteins in a single band. Gel bands were reduced with 10 mM DTT, alkylated in 50 mM iodoacetamide, and digested overnight at 37°C with 80 ng/mL of modified trypsin (Promega, Madison, WI, USA). The resulting tryptic peptides were extracted by incubation in 12 mM ammonium bicarbonate pH 8.8 and later, 0.5% trifluoroacetic acid (TFA). TFA was added to a final concentration of 1% and the peptides were desalted onto C18 Oasis-HLB cartridges and dried-down for further analysis. For stable isobaric labeling, the tryptic peptides were dissolved in triethylammonium bicarbonate (TEAB) buffer, and the peptide concentration was determined by measuring amide bonds with the Direct Detect system (Millipore, Billerica, MA, USA). Equal amounts of each peptide sample were labeled using the 8-plex iTRAQ Reagents Multiplex Kit (Applied Biosystems, Foster City, CA, USA) according to manufacturer › s protocol. Samples were then concentrated in a Speed Vac, desalted onto C18 Oasis-HLB cartridges and dried-down for further analysis. To increase proteome coverage, iTRAQ-labeled samples were fractionated by mixed-cation exchange chromatography (Oasis HLB-MCX columns) into six fractions, and analyzed by LC-MS/MS on an Orbitrap Elite mass spectrometer (Thermo Fisher Scientific, Waltham, MA, USA) using a C-18 reversed phase nano-column (75 μm internal diameter × 50 cm, 2 μm particle size, Acclaim PepMap RSLC, 100 C18; Thermo Fisher Scientific) in a continuous acetonitrile gradient consisting of 0–30% B in 360 min, 50–90% B in 3 min (A = 0.1% formic acid; B = 90% acetonitrile, 0.1% formic acid). A flow rate of 200 nL/min was used to elute peptides from the nano-column to an emitter nanospray needle for real time ionization and peptide fragmentation. An enhanced FT-resolution spectrum (resolution = 70,000) followed by the MS/MS spectra from the 15 most intense parent ions were analyzed along the chromatographic run. Dynamic exclusion was set at 40 s. For peptide identification, all spectra were analyzed with Proteome Discoverer (1.4.0.29, Thermo Fisher Scientific) using SEQUEST-HT (Thermo Fisher Scientific). For database searching at the Uniprot database, containing all sequences from human and cRAP contaminants (March 06, 2013; 70024 entries), the parameters selected were as follows: trypsin digestion with 2 maximum missed cleavage sites, precursor and fragment mass tolerances of 2 Da and 0.02 Da, respectively, carbamidomethyl cysteine, iTRAQ modifications at N-terminal and Lys residues as fixed modifications, and methionine oxidation as dynamic modification. Peptide identification was validated using the probability ratio method [46]; false discovery rate (FDR) was calculated using inverted databases and the refined method [47], with an additional filtering for precursor mass tolerance of 15 ppm [48]. Protein quantification from reporter ion intensities and statistical analysis of quantitative data to identify significant proteins were performed using QuiXoT based on a statistical model [49]. We applied stringent criteria, considering differentially expressed those proteins identified with more than three peptides, and log2-ratios expressed in the form of the standardized variables (Zq) ≥ 1.4 (absolute value) with *p*-values ≤ 0.05, with Zq denoting the mean of the two replicates *versus* the control group.

### Systems biology analysis

Identified proteins were compared to the entries in the ExoCarta database, a repository comprising proteins previously identified in exosomes [50]. Expression and localization of proteins in renal tubule and glomeruli were assigned by means of Human Protein Atlas [51] and scientific literature. PANTHER (Protein ANalysis THrough Evolutionary Relationships) database [52] and DAVID (6.7) [53] were used for protein functional classification. Protein pathway analysis was carried out using STRING 9.0 software (highest confidence, MCL clustering tool) [54], a database of known and predicted protein interactions.

### Target protein analysis (selected reaction monitoring (SRM)-LC-MS/MS)

Urine samples from the 40 non-diabetic hypertensive patients under chronic RAS suppression and 14 healthy subjects (including those analyzed by iTRAQ) were used for SRM-LC-MS/MS analysis. All individuals have been included in the iTRAQ analysis, but samples were analyzed by SRM individually, not including pools. Theoretical SRM transitions were designed using Skyline (1.1.0.2905) [55] and manually inspected. Protein specificity was confirmed by protein BLAST and proteotypic peptides were selected for SRM analysis (Supplementary Table 4). Protein extracts were obtained from exosomes isolated from individual urine samples. In total, 30 μg of protein per individual sample was digested and peptide mixtures were analyzed by SRM-LC-MS/MS as described [16, 45, 56] using a 6460 Triple Quadrupole LC/MS/MS (Agilent Technologies Inc., Santa Clara, CA, USA). Briefly, peptide separation was performed on a ProtID Zorbax 300B-C18-5 μm chip with a 43 × 0.075-mm analytical column and a 40 nL enrichment column (Agilent Technologies). Two microliters of sample was injected at 3 μL/min and separation took place at 0.4 μL/min in a continuous acetonitrile gradient: 70% B at 10 min, 95% B at 12 min until 14.2 min and 0% B for 2 min (B = 0.1% formic acid in acetonitrile). The system was controlled by Mass Hunter LC–MS Acquisition Software (v4.01). The mass spectrometer was operated in positive ion mode with capillary voltage of 1970 V, 325°C source gas temperature, and 5 L/min source gas flow. The fragmentor was set to 130 V, dwell time to 20 ms, delta EMV to 600 V, and collision energy was optimized for each SRM transition. For calculation of statistically significant differences in protein levels between groups, the ROUT method was applied to detect outliers based on the FDR, setting *Q* value to 5%. The Kruskal-Wallis test with Dunn's multiple comparisons pos*t*-test and 95% confidence level was then performed. Analyses were performed using GraphPad Prism 6 (6.01) software (GraphPad Prism Inc., La Jolla, CA, USA) and Metaboanalyst web server (3.0).

## SUPPLEMENTARY MATERIALS FIGURE AND TABLES





## Abbreviations

BP: blood pressure
RAS: renin-angiotensin system
iTRAQ: Isobaric tags for relative and absolute quantification
LC-MS/MS: liquid chromatography coupled with tandem mass spectrometry
N: normoalbuminuria
dnA: *de novo* albuminuria
MHA: maintained high albuminuria
SRM: selected reaction monitoring
AUC: area under the curve
GO: gene ontology
GAG: glycosaminoglycan
GBM: glomerular basement membrane
PTECs: proximal tubular epithelial cells
DTT: dithiothreitol
TEAB: triethylammonium bicarbonate
FDR: false discovery rate
