# Supplementary material for: Urinary exosomes reveal protein signatures in hypertensive patients with albuminuria
Source: Oncotarget. 2017 May 11;8(27):44217–31. doi: 10.18632/oncotarget.17787 (PMC5546475; doi:10.18632/oncotarget.17787)
Supplement: Supplementary file 1 [file oncotarget-08-44217-s001.pdf]

Urinary exosomes reveal protein signatures in hypertensive patients with albuminuria

Supplementary Materials

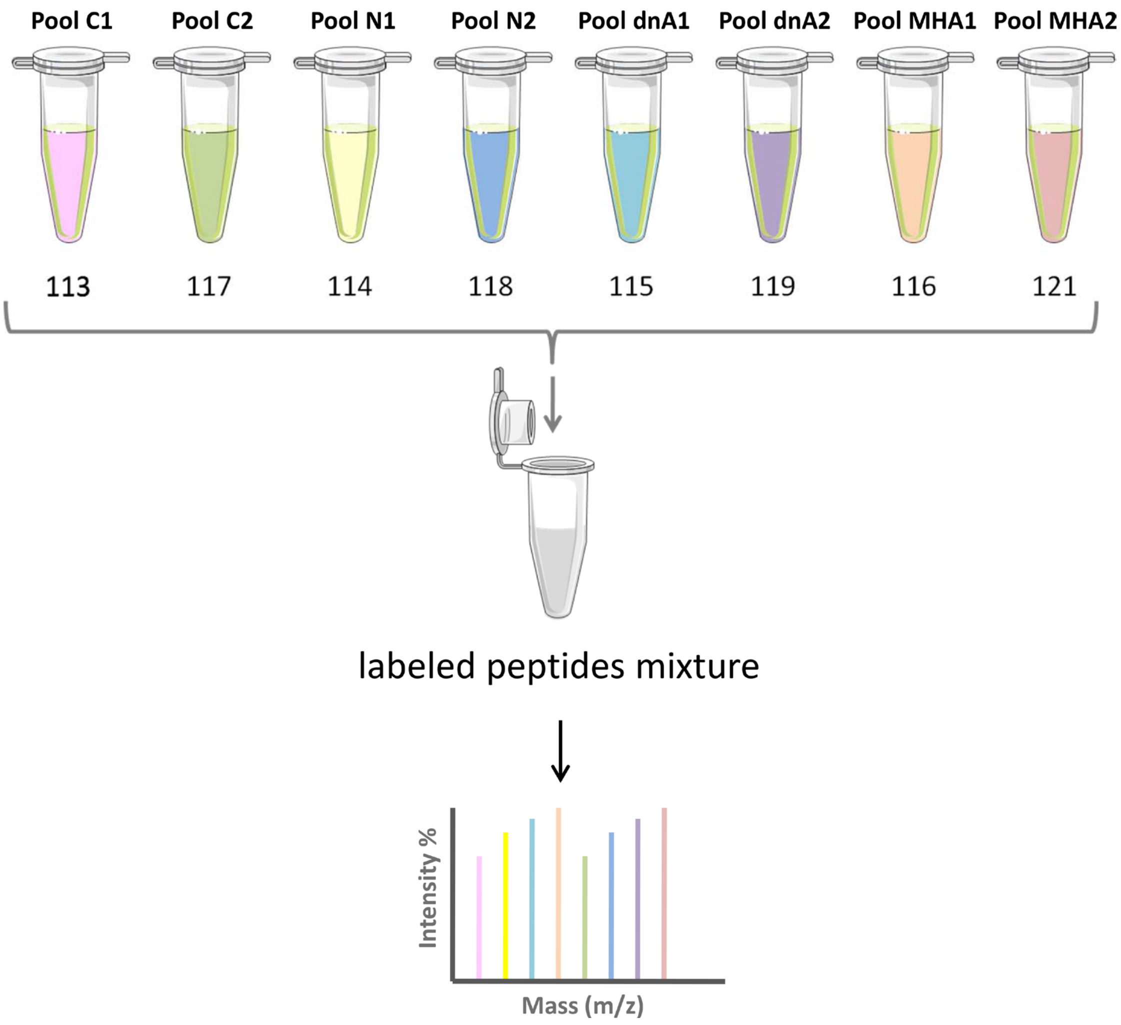

Supplementary Figure 1: Experimental design for iTRAQ labeling. Each pool is composed by 2–4 individual samples. Two biological replicates were analyzed per condition (C, N, dnA, MHA).

Supplementary Table 1: 487 proteins were identified with 4 or more peptides in urinary exosomal fraction from hypertensive patients under chronic suppression of RAS. See Supplementary_Table_1

Supplementary Table 2: Out of the 487 proteins identified in the exosomal fraction with 4 or more peptides, 100 proteins were here identified for the first time in urinary exosomes according to Exocarta repository. See Supplementary_Table_2

Supplementary Table 3: Proteins identified in our study without showing alteration in response to hypertension or albuminuria, and previously isolated form urinary exosomes and assigned to specific kidney parts

Fasta	Gene	Protein name	ID Peptides	Refs.		
**Bowman´s Capsule**						
O00592	PODXL	Podocalyxin	6	_(1)		_(4)
Podocytes						
Q9NP85	NPHS2	Podocin	4	_(2)		
P17927	CR1	Complement receptor type 1	6	_(2)		
Parietal cells						
P05787	KRT8	Keratin, type II cytoskeletal 8	7	_(3)		
**Proximal Convoluted tubule**						
P98164	LRP2	Low-density lipoprotein receptor-related protein 2	59	_(2)	_(1)	
O60494	CUBN	Cubilin	46	_(2)	_(1)	
P15144	ANPEP	Aminopeptidase N	55	_(1)		
O96009	NAPSA	Napsin-A	4	_(1)		
**Loop of henle**						
P07911	UMOD	Uromodulin	44	_(3)	_(1)	
Q9H6S3	EPS8L2	Epidermal growth factor receptor kinase substrate 8-like protein 2	7	_(3)		
Q8TE68	EPS8L1	Epidermal growth factor receptor kinase substrate 8-like protein 1	6	_(3)		
**Distal convoluted tubule**						
Q8N271	PROM2	Prominin-2	6	_(3)		
P55017	SLC12A3	Solute carrier family 12 member 3	12	_(3)	_(1)	_(5)
**Collecting Duct**						
P38606	ATP6V1A	V-type proton ATPase catalytic subunit A	14	_(3)		
P15313	ATP6V1B1	V-type proton ATPase subunit B, kidney isoform	9	_(3)		
Q93050	ATP6V0A1	V-type proton ATPase 116 kDa subunit a isoform 1	5	_(3)		
Q9UI12	ATP6V1H	V-type proton ATPase subunit H	4	_(3)		
Q9HBG4	ATP6V0A4	V-type proton ATPase 116 kDa subunit a isoform 4	4	_(3)		
**Bladder**						
O00526	UPK2	Uroplakin-2	4	_(2)		
P08727	KRT19	Keratin, type I cytoskeletal 19	18	_(3)		

REFERENCES

1. Dear JW, Street JM, Bailey MA. Urinary exosomes: a reservoir for biomarker discovery and potential mediators of intrarenal signalling. Proteomics. 2013; 13:1572–80.
2. Musante L. Use and isolation of urinary exosomes as biomarkers for diabetic nephropathy. Front Endocrinol (Lausanne). 2014; 5:149.
3. Jayachandran M, Lugo G, Heiling H, Miller VM, Rule AD, Lieske JC. Extracellular vesicles in urine of women with but not without kidney stones manifest patterns similar to men: a case control study. Biol Sex Differ. 2015; 6:2.
4. Raj DA, Fiume I, Capasso G, Pocsfalvi G. A multiplex quantitative proteomics strategy for protein biomarker studies in urinary exosomes. Kidney Int. 2012; 81:1263–72.
5. Esteva-Font C, Wang X, Ars E, Guillén-Gómez E, Sans L, González Saavedra I, Torres F, Torra R, Masilamani S, Ballarín JA, Fernández-Llama P. Are sodium transporters in urinary exosomes reliable markers of tubular sodium reabsorption in hypertensive patients? Nephron Physiol. 2010; 114:25–34.

Supplementary Table 4: SRM-LC-MS/MS analysis conditions

fasta	protein	description Gene	TREND	Precursor peptide sequence	Precursor —>Fragment (m/z)	Collision energy	Fragmentor (V)	Dwell time (ms)
Q6UX06	OLFM4_HUMAN	Olfactomedin-4 (OLFM4)	↑	K.ESFGGSSEIVDQLEVEIR.N	665.33 —> 675.31	19.2	130	20
P01008	ANT3_HUMAN	Antithrombin-III (SERPINC1)	↑	K.SLTFNETYQDISELVYGAK.L	726.69 —> 779.42	21.4	130	20
P05164	PERM_HUMAN	Myeloperoxidase (MPO)	↓	R.AADYLHVALDLLER.K	799.93 —>841.42	25.8	130	20
